# Performance Measures and Plasma Biomarker Levels in Patients with Multiple Sclerosis after 14 Days of Fampridine Treatment: An Explorative Study

**DOI:** 10.3390/ijms25031592

**Published:** 2024-01-27

**Authors:** Maria Thorning, Kate Lykke Lambertsen, Henrik Boye Jensen, Lars Henrik Frich, Jonna Skov Madsen, Dorte Aalund Olsen, Anders Holsgaard-Larsen, Helle Hvilsted Nielsen

**Affiliations:** 1 Department of Neurology, Odense University Hospital, J.B. Winsloews Vej 4, 5000 Odense C, Denmark; klambertsen@health.sdu.dk (K.L.L.); helle.hvilsted.nielsen@rsyd.dk (H.H.N.); 2 Orthopaedic Research Unit, Department of Clinical Research, University of Southern Denmark, Campusvej 55, 5230 Odense M, Denmark; ahlarsen@health.sdu.dk; 3 Department of Neurobiology Research, Department of Molecular Medicine, University of Southern Denmark, J.B. Winsloews Vej 21, st., 5000 Odense C, Denmark; lars.henrik.frich@rsyd.dk; 4 BRIDGE—Brain Research—Inter Disciplinary Guided Excellence, Department of Clinical Research, University of Southern Denmark, J.B. Winsloews Vej 19.3, 5000 Odense C, Denmark; 5 Department of Brain and Nerve Diseases, Lillebaelt Hospital, University Hospital of Southern Denmark, Sygehusvej 24, 6000 Kolding, Denmark; henrik.boye.jensen@rsyd.dk; 6 Department of Orthopaedics, Hospital Soenderjylland, Kresten Philipsens Vej 15, 6200 Aabenraa, Denmark; 7 Department of Regional Health Research, University of Southern Denmark, J.B. Winsloews Vej 19.3, 5000 Odense C, Denmark; jonna.skov.madsen@rsyd.dk; 8 Department of Biochemistry and Immunology, Lillebaelt Hospital, University Hospital of Southern Denmark, Beriderbakken 4, 7100 Vejle, Denmark; dorte.aalund.olsen@rsyd.dk; 9 Department of Orthopaedics and Traumatology, Odense University Hospital, J.B. Winsloews Vej 4, 5000 Odense C, Denmark

**Keywords:** cytokines, neurofilament light chain, glial fibrillary acidic protein, inflammation, performance measures, physical activity

## Abstract

Peripheral cytokine levels may serve as biomarkers for treatment response and disease monitoring in patients with multiple sclerosis (pwMS). The objectives were to assess changes in plasma biomarkers in PwMS after 14 days of fampridine treatment and to explore correlations between changes in performance measures and plasma biomarkers. We included 27 PwMS, 14 women and 13 men, aged 52.0 ± 11.6 years, with a disease duration of 17 ± 8.5 years, and an Expanded Disability Status Scale of 6 [IQR 5.0/6.5]. Gait and hand function were assessed using performance tests completed prior to fampridine and after 14 days of treatment. Venous blood was obtained, and chemiluminescence analysis conducted to assess plasma cytokines and neurodegenerative markers. All performance measures demonstrated improvements. Biomarkers showed decreased tumor necrosis factor (TNF) receptor-2 levels. Associations were found between change scores in (i) Six Spot Step Test and Interleukin (IL)-2, IL-8, and IL-17 levels; (ii) timed 25-foot walk and interferon-γ, IL-2, IL-8, TNF-α, and neurofilament light levels, and (iii) 12-Item Multiple Sclerosis Walking Scale and IL-17 levels. The associations may reflect increased MS-related inflammatory activity rather than a fampridine-induced response or that a higher level of inflammation induces a better response to fampridine.

## 1. Introduction

Multiple sclerosis (MS) is a chronic autoimmune and demyelinating disorder of the central nervous system (CNS). It is characterized by inflammation and neurodegeneration that lead to a wide range of motor, sensory, and cognitive dysfunctions, and it constitutes the main cause of nontraumatic disability in young adults [1]. As up to 76% of people with MS require ambulatory aid during the course of the disease [2], gait limitations are the main cause of disability. These are often considered the primary challenge of the condition as they influence the quality of life [3]. Therefore, there is a demand for both interventions to improve gait as well as valid and reliable measurements to monitor treatment responses.

Fampridine is a medical drug that improves gait performance in a subset of pwMS [4,5,6,7,8]. By blocking voltage-dependent potassium channels, fampridine enhances the signal conduction in demyelinated axons [9]. This improves gait through increased walking speed [4,5,8], higher endurance [4], and better self-perceived gait function [6,7]. These, in turn, increase quality of life and daily activity levels [10].

Research suggests that physical activity can reduce the level of proinflammatory cytokines and increase the level of anti-inflammatory cytokines, indicating that consistent physical exercise has the potential to reverse chronic inflammation [11]. Cytokines are often useful as biomarkers as they provide information about biological and pathogenic processes and can help in monitoring treatment effects and predicting clinical outcomes [12]. Treatment with fampridine may indirectly affect cytokine biomarkers through increased activity in pwMS. However, to the best of our knowledge, fampridine-induced changes in biomarkers after 14 days of treatment have not previously been explored.

It is believed that MS commences in the immune system and that inflammatory responses orchestrated by cytokines are central processes in autoimmune attacks [13]. Cytokines are small proteins that permit communication between cells and the surrounding tissue and are a key factor in the coordination of immune factors [13]. The classes of cytokines that are most important in MS are the interleukins (ILs), interferons (IFNs), and tumor necrosis factor (TNF) with its receptors TNFR1 and TNFR2 [13]. Previous studies have shown abnormal cytokine levels in the blood and cerebrospinal fluid (CSF) of pwMS compared with controls [14,15,16,17,18]. Additionally, markers for neuronal damage and glial activation, such as neurofilaments (NF) and glial fibrillary acidic protein (GFAP) [19], correlate with disease severity, activity, and duration [20] and have prognostic value [21], making them potential biomarkers in MS.

Previous studies involving subgroups of the participants included in the present study revealed significant fampridine-induced changes in timed 25-foot walk (T25FW), Six Spot Step Test (SSST), 2-minute walk test (2MWT), 12-Item Multiple Sclerosis Walking Scale (MSWS-12), and Nine-Hole Peg Test (9HPT) [22,23]. Plasma biomarkers could provide a further option for monitoring treatment responses following fampridine treatment.

Thus, the objectives of the current study were (i) to assess potential changes in relevant plasma biomarkers in pwMS after 14 days of fampridine treatment and (ii) to explore the correlations between potential fampridine-induced changes in biomarker levels and changes in performance measures. We estimated blood levels of selected proinflammatory cytokines (IL-2, IL-8, IL-17, IFN-γ, and TNF), an anti-inflammatory cytokine (IL-4), the TNF receptors TNFR1 and TNFR2, and markers of axonal damage (NF light chain (NFL) and astroglial activation (soluble GFAP)) at baseline and 14 days after initiation of fampridine treatment. We then correlated hypothesized changes in these biomarkers to potential changes in performance measures (T25FW, SSST, 2MWT, MSWS-12, and 9HPT).

## 2. Results

**Figure 1:**
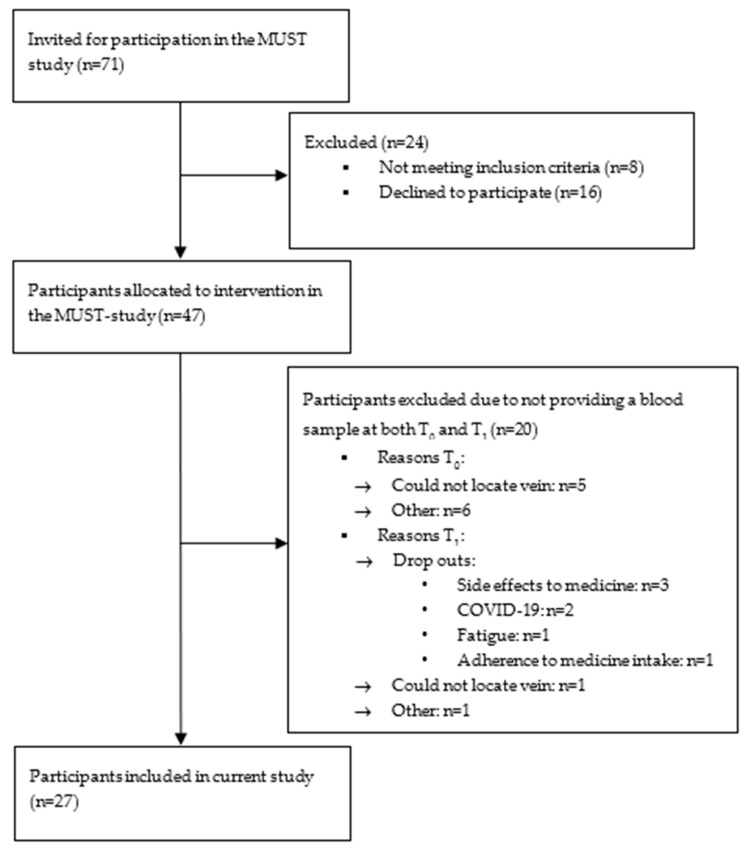
Flowchart of participant inclusion.

Of the 71 eligible patients invited to participate in the larger cohort study (MUST), 16 patients declined, 8 did not meet the inclusion criteria, and 20 did not provide blood samples at both visits (Figure 1). Thus, 27 participants were included in the current analyses. The mean age was 52.0 ± 11.6 years, 51.9% were women, the mean disease duration was 17 ± 8.5 years, and the median EDSS was 6 [IQR 5.0/6.5]; see Table 1.

At T_0_, 3.7% of patients had missing data for performance measures (MSWS-12) and 3.7% to 18.5% for biomarkers. At T_1_, 3.7% to 7.4% had missing data for performance measures (2MWT and MSWS-12), and 3.7% to 18.5% for biomarkers. An overview of missing data is presented in Supplementary Table S1a,b.

In accordance with our previous results for subgroups of participants also included in the present study [22,23], we saw significant improvements between T_0_ and T_1_ in all included performance measures (T25FW: −0.70, *p* = 0.0012; SSST: −1.80, *p* = 0.0003; 2MWT: 14.97, *p* = 0.0001; 9HPT: −1.90, *p* = 0.0048; MSWS-12: −16.25, *p* < 0.0001) (Table 2a). Among the biomarkers, however, only TNFR2 levels showed significant group mean changes (a decrease from T_0_ to T_1_) (−408.34, *p* = 0.0466) (Table 2b). Nonetheless, all included biomarkers showed significant individual differences, see Table 2b and Supplementary Figure S1.

**Figure 2:**
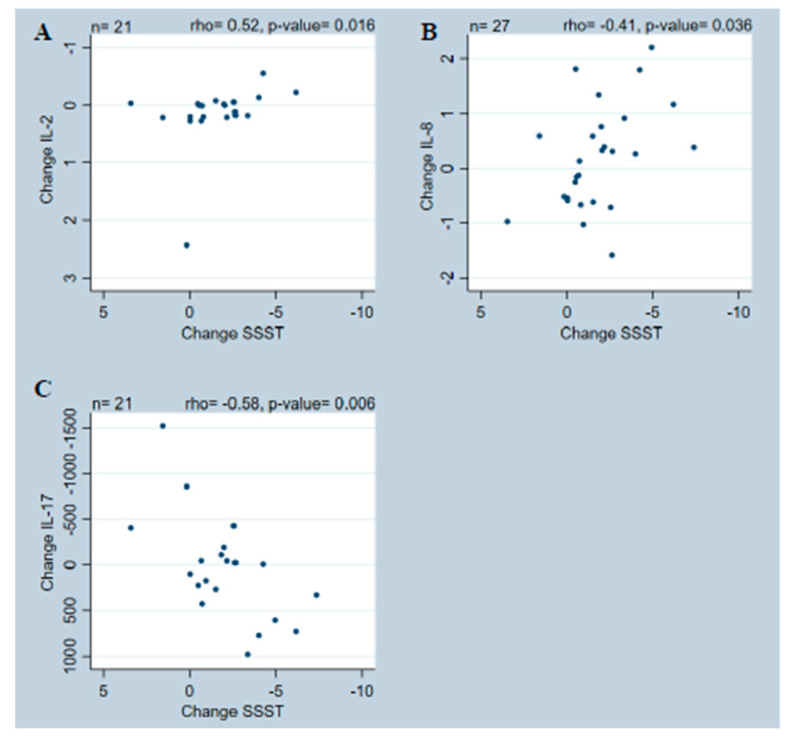
Significant correlations between change in SSST and (**A**) IL-2, (**B**) IL-8, and (**C**) IL-17. IL—Interleukin; SSST—Six Spot Step Test.

**Figure 3:**
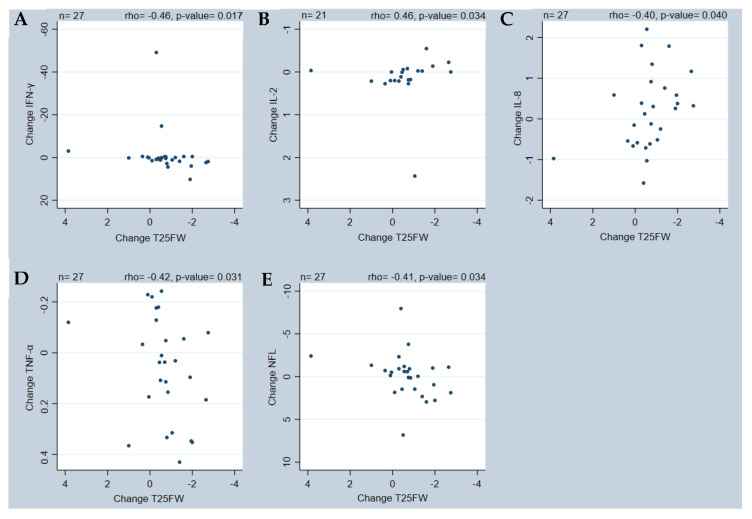
Significant correlations between change in T25FW and (**A**) IFN-γ, (**B**) IL-2, (**C**) IL-8, (**D**) TNF-α, and (**E**) NFL. IFN-γ—Interferon gamma; IL—Interleukin; NFL—Neurofilament light; T25FW—timed 25-foot walk; TNF-α—tumor necrosis factor alpha.

**Figure 4:**
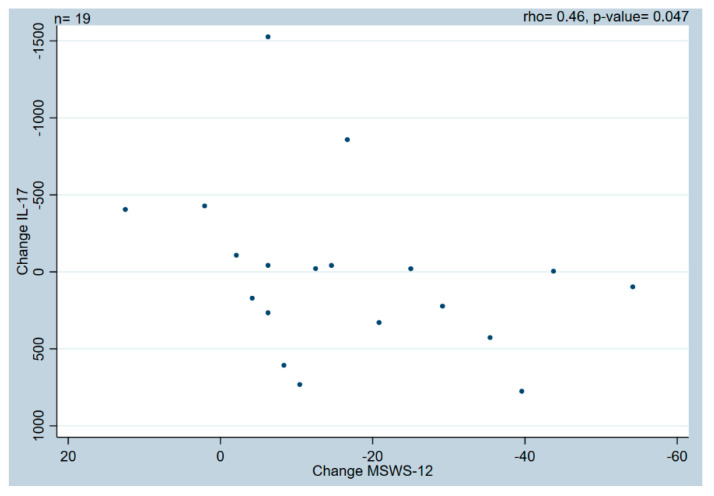
Significant correlations between change in MSWS-12 and IL-17. IL—Interleukin; MSWS-12—12-Item Multiple Sclerosis Walking Scale.

Therefore, we examined possible correlations between changes in performance measures and changes in biomarkers. We found (i) the associations between changes in SSST and changes in IL-2 levels (rho = 0.52, *p* = 0.0162) (Figure 2A) and IL-17 (rho = −0.58, *p* = 0.0058) (Figure 2C) were moderate to good, and fair between changes in SSST and IL-8 (rho = −0.41, *p* = 0.0357) (Figure 2B). (ii) The associations were fair between changes in T25FW and changes in IFN-γ (rho = −0.46, *p* = 0.0168) (Figure 3A), IL-2 (rho = 0.46, *p* = 0.0339) (Figure 3B), IL-8 (rho = −0.40, *p* = 0.0398) (Figure 3C), TNF (rho = −0.42, *p* = 0.0309) (Figure 3D), and NFL (rho = −0.41, *p* = 0.0343) (Figure 3E) levels. And (iii) the association was fair between changes in MSWS-12 and changes in IL-17 levels (rho = 0.46, *p* = 0.0473) (Figure 4). For all results on correlation data, see Supplementary Table S2.

## 3. Discussion

A practical clinical approach was employed in this study, in which fampridine was given to participants during a startup period (as part of the larger MUST study), and we assessed its potential clinical benefits on physical performance measures, biomarkers, and their hypothetical association. The sample included in this study comprised both fampridine responders and nonresponders, acting as their own controls.

Consistent with our previously reported results on data from subgroups of the MUST study [22,23,24], 14 days of fampridine treatment resulted in significant mean improvements in all included performance measures in pwMS. Among the biomarkers, however, only TNFR2 levels showed a significant mean decrease. TNFR2 is known to have an important role in both the innate immune system and the remyelinating processes [25,26]. However, the biological importance of the decrease in TNFR2 levels remains to be investigated.

To the best of our knowledge, no previous studies have explored the effect of fampridine on inflammation in pwMS. Although we have no data on physical activity, previous studies have demonstrated that fampridine improves gait function [27] and that physical activity can alter the level of inflammatory cytokines [11]. Our results suggest that fampridine does not influence the quantity of physical activity to such a degree that it affects the levels of inflammatory markers in the blood of pwMS following 14 days of treatment. Even though pharmacokinetic studies have demonstrated that fampridine treatment is fully active in a two-week trial-period plasma where concentrations are reached within 3.75 h after the first dose with a half-life of 5.45 h [28], it is possible that a longer treatment period might have led to a different result. It is, therefore, a relevant topic for future research.

Although a significant group mean change was only found for TNFR2, we observed large individual differences in all the evaluated biomarkers. These variations are reflected in several of the correlations between fampridine-induced changes in performance measures and changes in levels of biomarkers, i.e., (i) associations between changes in SSST and changes in IL-2, IL-8, and IL-17 levels; (ii) associations between changes in T25FW and changes in IFN-γ, IL-2, IL-8, TNF, and NFL levels, and (iii) associations between changes in MSWS-12 and changes in IL-17 levels.

These associations indicate that participants who improved more in the SSST after 14 days of fampridine treatment also demonstrated a greater decrease in IL-2 levels but an increase in IL-8 and IL-17 levels. As pwMS generally display elevated IL-2 levels [17,29] and decreased IL-8 levels [17,18], this could be interpreted as an anti-inflammatory effect. This result was not supported by IL-17, however, which is generally considered a key inflammatory cytokine that is increased in pwMS [30,31,32]. Interestingly, a similar correlation was found with the patient-reported measure, where greater improvements in MSWS-12 correlated with higher IL-17 levels.

Similar divergent results were found in the correlations for T25FW, where participants who improved more in the T25FW displayed a greater decrease in IL-2 levels as well as a greater increase in IFN-γ, IL-8, TNF, and NFL levels. As mentioned earlier, the IL-2 and IL-8 results could be interpreted as an anti-inflammatory effect, whereas IFN-γ, TNF, and NFL are increased in pwMS and considered indicative of MS activity [14,16,17,32,33,34]. Taken together, these results underline the complexity of the immunopathogenesis in MS and indicate the need for careful interpretation of results from small populations with short observation periods, as in the current study.

Although our demonstrated correlations are interesting, they should be interpreted with caution. Several significant correlations include 0 in their 95% Bca, so their robustness may be questionable. Inspection of the correlation graphs revealed several outliers in the changes for IFN-γ, IL-2, IL-17, T25FW, and 2MWT. These could potentially alter the conclusion of the correlations, although post hoc analysis indicated the reported conclusions to be solid and not influenced by outliers. The evaluation period of 14 days is short, and the results could just reflect the heterogeneity of MS with its fluctuating levels of inflammation. Thus, higher inflammatory activity might induce a better response to treatment with fampridine. Similarly, the changes observed in the biomarkers for individual participants may be an expression of increased inflammatory activity generated by MS rather than an actual effect of the fampridine. This may be supported by the observed association between better T25FW scores and increases in NFL levels since NFL is an expression of neurodegeneration [33] and, therefore, usually increases with disease activity. Previous studies have shown contradictory results regarding the influence of the active drug in fampridine (4-aminopyridine) on inflammatory cells and its response in in vitro and animal models [35]. Some studies reported a positive impact of 4-aminopyridine on the inflammatory response, but they used higher doses of fampridine than our study and had treatment durations of up to 90 days (compared with our 14-day period) [35].

We included a wide selection of well-known biomarkers relevant to MS. Biomarkers are potentially valuable for treatment and disease monitoring in pwMS because of their pro- and anti-inflammatory properties [17]. The current study shows that changes in both IL-2 and IL-8 levels are significantly related to changes in SSST and T25FW, so these might be better candidates for further investigations of biomarkers for changes in performance in pwMS. The changes in IL-17 levels showed both positive and negative associations with changes in performance measures, making it difficult to draw any conclusions about the relevance of this specific biomarker. The inclusion of additional biomarkers or analyses of specific cellular inflammation markers might have provided further results, but it is unlikely since a large selection of biomarkers already was included in the current study.

All significant correlations between changes in T25FW and MSWS-12 and changes in biomarkers were only fair, and their relevance may be questionable. However, T25FW combined with SSST were the performance measures that were most efficiently associated with changes in biomarkers after 14 days of fampridine treatment in the current study. Therefore, these performance measures should be included in future research on this topic.

### Strength and Limitations

We believe this is the first study to examine potential fampridine-induced changes in biomarkers in pwMS. As such, it contributes novel and valuable information about the influence of fampridine on biomarkers, in addition to its established impact on gait function in pwMS.

The current study has some methodological limitations. It is an explorative study, and its pragmatic clinical approach entailed a small sample size and the inclusion of both fampridine responders and non-responders without a control group. Thus, the generalizability, statistical power, and validity of the study findings may be limited, leading to potential inaccuracies and errors. Furthermore, the lack of randomization reduces the ability to investigate causation and raises concerns about potential placebo effects, performance bias, and recall bias that could impact the results. Nevertheless, the current study, with its explorative character, is a hypothesis-generating study. Previous studies have tested fampridine against placebo, and the substantial amount of literature demonstrating the positive impact of fampridine on gait function over a 14-day trial period [5,6,36,37] provides support for the current findings. Additionally, the cytokine levels in our participants compared to cytokine levels in healthy controls in a study from our own lab using the same type of test kits and the same time frame as our study [38] verify the results of altered cytokine levels in our sample of pwMS compared with healthy controls.

Our analyses were conducted on complete data and analyzed as observed. Because of the low sample size, we did not consider multiple imputation or inverse probability weighting to be viable strategies for addressing missing data. Joint modeling of outcomes between visits using mixed models was also investigated, but as assumptions of Gaussian distribution were found to be violated, this approach was not applied.

We measured biomarkers in plasma and not CSF. The proximity of CSF to inflammatory lesions in the CNS theoretically makes it a better sample to reflect the relevant inflammatory process, but the flow pattern of the CSF makes it unlikely to accurately reflect inflammation in the supratentorial region where most MS-related inflammation occurs [39]. Moreover, collecting CSF is an invasive procedure, and its advantages over other markers collected from, e.g., blood, remain unclear [39].

As we did not measure physical activity directly, our results do not determine whether the associations observed between changes in performance measures and changes in biomarkers reflect a fampridine-induced increased activity level that could reduce the levels of proinflammatory markers in the blood. Furthermore, measures of mental health could have been an interesting angle.

## 4. Materials and Methods

### 4.1. Participants and Setting

The current exploratory cohort study is a sub-study of a larger prospective observational cohort study (the MUST study) (Trial registration: ClinicalTrials.gov NCT03847545) described previously [22,23,24]. The MUST study was conducted from December 2018 to October 2021 at Odense University Hospital, Odense, Denmark. The present sub-study is reported according to the Strengthening the Reporting of Observational Studies in Epidemiology (STROBE) statement for reporting observational studies [40].

The MUST study was approved by the National Committee on Health Research Ethics (S-20170203), reported to the Danish Data Protection Agency (2012-58-0018), and conducted in accordance with the Declaration of Helsinki. Prior to inclusion, all participants received oral and written information and provided written informed consent.

Recruitment of eligible participants was conducted as described previously [22,23,24] from the outpatient MS Clinic at Odense University Hospital, Odense, Denmark, and consisted of pwMS in a stable disease state defined as no relapse within 60 days of inclusion and no MRI activity within the last year. Both patients with and without immunomodulatory treatment were included (Table 1), but the choice of treatment remained unchanged within the previous 60 days.

Inclusion criteria furthermore included a clinical diagnosis of MS according to the McDonald criteria [41], age > 18 years, an Expanded Disability Status Scale score (EDSS) between 4 and 7, and attainment of the guidelines for receiving fampridine according to clinical symptoms and neurological status. Exclusion criteria were diagnosed epilepsy, cancer within the last five years, clinically significant systemic disease, change in immunomodulatory treatment within the last 60 days, MS attacks or an acute decrease in functional capacity within the past 60 days, and concurrent treatment with cimetidine, carvedilol, propranolol, or metformin [22,23,24].

The participants completed a baseline visit (T_0_) and a follow-up visit after 14 (±1) days (T_1_). The intervention between the two visits consisted of 10 mg fampridine^®^ (Biogen, Cambridge, MA, USA) twice daily [22,23]. At both visits, participants completed the included performance measures, gave a blood sample, and a urine screening for cystitis was performed since this is the most common infection in pwMS [42]. None of the participants showed or declared any signs of infections. Walking aids were used as required and kept identical for the individual performance measures at T_0_ and T_1_. At T_0_, baseline characteristics were collected, including age, gender, disease duration, sub-diagnosis, medical treatment, and EDSS.

### 4.2. Outcome Measures

#### 4.2.1. Performance Measures

The execution of the included performance measures in the MUST study has been described previously [22,23,24]. In short, we included the following variables in the current study: (i) The T25FW, which assesses short-distance walking speed and was completed in agreement with the guidelines for the Multiple Sclerosis Functional Composite [43]; (ii) The SSST, evaluating gait ambulatory function through timed criss-cross walking across a rectangular course, while kicking five blocks out of circles marked on the floor [44]; (iii) The 2MWT, a measure of walking endurance [45] that was performed with the participants walking laps on a 20-m lane, turning around cones at each end, for two minutes, while the total distance travelled was recorded; (iv) The MSWS-12, a patient-reported outcome measure that evaluates self-perceived gait impairments due to MS during the past two weeks with the total score transformed to a scale from 0 to 100 (minimum to maximum walking disability) [46]; and (v) the 9HPT, a measure of upper extremity function [47] that was performed according to the guidelines for the Multiple Sclerosis Functional Composite [43] and included for the dominant side.

#### 4.2.2. Plasma Biomarkers

Venous blood was obtained using 2 × 4 mL BD Vacutainer EDTA tubes. Within two hours of sampling, plasma was centrifuged at 2000× *g* for 10 min at 20° (room temperature), aliquoted into 2 mL Sarstedt polypropylene tubes, and stored at −80° until further analyses. In this manuscript, the term “biomarkers” refers to plasma biomarkers unless otherwise stated.

##### Chemiluminescence Analysis

Plasma concentrations of TNF, IFN-γ, IL-2, IL-4, and IL-8 were measured using a V-PLEX Custom Human Proinflammatory Panel 1 kit (K151A9H), plasma concentrations of IL-17 using a Human IL-17A S-PLEX kit (K151C3), and plasma concentrations of TNFR1 and TNFR2 using Human TNFR-I ultrasensitive (K151BIC) and Human TNFR-II ultrasensitive (K151BJC) kits (All from Mesoscale Discovery (Meso Scale Diagnostics, Rockville, MD, USA)). Samples were diluted in Diluent-41 and analyzed in duplicate according to the manufacturer’s instructions. Analysis was performed on a SECTOR Imager 6000 plate reader and MSD Discovery Workbench software 4.0 (Mesoscale Discovery, Meso Scale Diagnostics, Rockville, MD, USA) as previously described [48,49]. Sample replicates with coefficient of variation (CV) values > 25% in individual analyses were excluded. Lower levels of detection (LLOD) were: IL-2 = 0.045–0.058 pg/mL; IL-4 = 0.016–0.025 pg/mL; IL-8 = 0.023–0.045 pg/mL; IL-17 = 49.0–87.8 pg/mL; IFN-γ = 0.593–0.951 pg/mL; TNF = 0.057–0.153 pg/mL; TNFR1 = 0.264–1.900 pg/mL; TNFR2 = 0.698–2.140 pg/mL. For protein levels below LLOD, a value of 0.5 LLOD was used for statistical analysis. Cytokine measurements were performed by a technician blinded to the clinical data.

##### Single-Molecule Array Analysis

Plasma concentrations of NFL and GFAP were analyzed at the Department of Biochemistry and Immunology, Lillebaelt Hospital, Vejle, using the commercially available 2-plex assay for the Single-Molecule Array Analysis (Simoa) HD-X analyzer (Quanterix, Billerica, MA, USA). The samples were diluted four times in buffer included in the kit and analyzed as single determinations. The operator was blinded to clinical data. Assay quality controls—two provided by the manufacturer and two in-house prepared serum pools—were included in each run to evaluate and monitor assay performance over time. The analytical CV% were <13%.

### 4.3. Statistical Methods

The Gaussian distribution of the included data was examined by visual inspection of normal probability plots combined with the Shapiro–Wilk test. Descriptive statistics are reported using numbers (*n*) and percentages (%), mean values and standard deviation (SD), or median values and interquartile range (IQR). All analyses were performed on complete cases and analyzed as observed. Potential changes between T_0_ and T_1_ were investigated using Student’s paired *t*-test with mean change and confidence intervals (95% CI) as well as Wilcoxon signed rank test, including median with nonparametric bootstrapping to obtain bias-corrected, accelerated bootstrap confidence intervals (95% BCa).

The included sample size was small, and prior to the analyses, we had no hypotheses for the specific functional association between the change scores of the performance measures and the biomarkers. Scatter plots were generated and visually inspected to evaluate any potential linear relationship between the individual variables [50], demonstrating no clear assumptions of linearity. Consequently, the correlations between the included performance measures and the biomarkers used Spearman’s rank–order correlation coefficient *ρ* (rho) with nonparametric bootstrapping to attain 95% BCa.

The strength of correlation coefficients was interpreted as follows: little to no relationship (0.00 to 0.25), fair relationship (0.25 to 0.50), moderate to good relationship (0.50 to 0.75), and good to excellent relationship (>0.75) [51]. The statistical analyses were performed in Stata/BE 17.0 (StataCorp LLC, College Station, TX, USA).

## 5. Conclusions

As reported previously [22,23], significant mean improvements in performance measures were observed after 14 days of fampridine treatment. However, the mean changes seen in biomarkers in the current study suggest that this treatment period may be too short to influence the quantity of physical activity that could affect levels of inflammatory markers.

The observed individual changes in biomarker levels, which were reflected in the associations between performance measures and biomarkers, could indicate an increased MS-related inflammatory activity rather than a fampridine-induced response. Nevertheless, the associations could also indicate that a higher level of MS-related inflammatory activity induces a better response to treatment with fampridine. Although the current reported results need further validation, further research into how biomarkers and levels of inflammation are influenced by fampridine treatment in pwMS is encouraged.

## Figures and Tables

**Table 1:** Baseline characteristics of participants (*n* = 27).

**Age, Years**	52.0 (11.6)
**Females, *n* (%)**	14 (51.9)
**Disease duration, years**	17.0 (8.5)
**Subdiagnosis, *n* (%):**	
Relapsing–remitting	11 (40.7)
Secondary progressive	11 (40.7)
Primary progressive	5 (18.5)
**Disease-modifying treatment, *n* (%):**	
Aubagio	3 (11.1)
Tecfidera	3 (11.1)
Tysabri	2 (7.4)
Lemtrada	1 (3.7)
Copaxone	2 (7.4)
Ocrevus	4 (14.8)
Rituximab	3 (11.1)
Methotrexate	1 (3.7)
No treatment	8 (29.6)
**EDSS**	6 [5.0/6.5]
**Patients using walking aids during the following tests, *n* (%):**	
T25FW	17 (47.2)
SSST	18 (50.0)
2MWT	23 (63.9)
**Smoking status, *n* (%):**	
No	21 (77.8)
Yes	5 (18.5)
Not answered	1 (3.7)
**Alcohol intake, units per week, *n* (%):**	
0	9 (33.3)
1–3	12 (44.4)
4–7	4 (14.8)
≥8	1 (3.7)
Not answered	1 (3.7)

Values are reported as mean (SD), median [IQR], or *n* (%). Abbreviations: 2MWT—2-minute walk test; EDSS—Expanded Disability Status Score; *n*—number; SD—standard deviation; SSST—Six Spot Step Test; T25FW—timed 25-foot walk.

**Table 2:** (**a**) Performance measures at baseline (T_0_) and 14 days follow-up (T_1_) in patients with MS before and after fampridine treatment. (**b**) Biomarkers in plasma at baseline (T_0_) and 14 days follow-up (T_1_) in patients with MS before and after fampridine treatment.

**(a)**						
**Performance Measures**	**Numbers (*n*)**	**T_0_**	**T_1_**	**Change [95% CI]**	**% Change**	***p*-Value**
**T25FW** (s)	27	7.05 [5.85/10.05]	6.35 [5.25/9.95]	−0.70 [−0.85; −0.30] *	−9.93	**0.0012**
**SSST** (s)	27	13.03 [9.58/20.63]	12.18 [8.85/16.63]	−1.80 [−2.70; −0.90]	−13.81	**0.0003**
**2MWT** (m)	26	111.29 (38.32)	126.76 (43.34)	14.97 [13.26; 21.64] *	13.45	**0.0001**
**9HPT** (s)	27	25.55 [22.45/30.10]	24.20 [21.10/28.95]	−1.90 [−2.80; −0.95] *	−7.44	**0.0048**
**MSWS-12** (points)	25	68.83 (17.23)	50.00 [41.67/72.92]	−16.25 [−22.93; −9.57]	−23.61	**<0.0001**
**(b)**						
**Biomarkers (pg/mL)**	**Numbers (*n*)**	**T_0_**	**T_1_**	**Change [95% CI]**		***p*-Value**
**IL-2**	21	0.35 [0.13/0.62]	0.33 [0.22/0.70]	0.01 [−0.03; 0.18] *		0.1981
**IL-4**	25	0.02 [0.01/0.05]	0.03 [0.01/0.04]	0.00 [0.01; 0.01] *		0.9662
**IL-8**	27	2.35 [1.76/3.13]	2.47 [1.55/3.66]	0.19 [−0.18; 0.57]		0.2967
**IL-17**	21	948.81 [540.56/1447.22]	1168.57 [763.19/1341.31]	45.55 [−210.55; 301.66]		0.7145
**IFN-γ**	21	3.36 [1.58/6.04]	3.02 [2.41/5.90]	0.31 [−0.02; 1.49] *		0.1363
**TNF-α**	27	1.12 [0.99/1.23]	1.15 [0.96/1.35]	0.06 [−0.02; 0.14]		0.1456
**TNF-R1**	26	1640.96 [1276.69/1903.46]	1495.61 [1126.99/1820.22]	−72.15 [−184.14; 39.85]		0.1966
**TNF-R2**	27	4862.52 [4272.24/5502.56]	4312.32 [3774.74/5675.94]	−408.34 [−810.02; −6.66]		**0.0466**
**NFL**	27	8.85 [7.04/12.09]	8.88 [7.22/12.04]	−0.51 [−0.91; 1.46] *		0.8288
**GFAP**	27	97.75 [61.26/135.52]	98.18 [77.16/133.23]	−0.77 [−9.91; 8.37]		0.8646

Data presented in table are reported on complete cases. Values at T_0_ and T_1_ are mean (SD) or median [IQR]. Change scores are mean [95% CI] if normally distributed or median [95% BCa] (followed by *) if non-normally distributed. Abbreviations: 2MWT—2-min walk test; 9HPT—Nine-Hole Peg Test; 95% BCa—bias-corrected, accelerated bootstrap confidence interval; GFAP—glial fibrillary acidic protein; IFN-γ—interferon gamma; IL—interleukin; IQR—interquartile range; m—meters; MSWS-12—12-Item Multiple Sclerosis Walking Scale; *n*—numbers; NFL—neurofilament light; s—seconds; SD—standard deviation; SSST—Six Spot Step Test; T_0_—baseline visit; T_1_—follow-up visit; T25FW—timed 25-foot walk; TNF-α—tumor necrosis factor-alpha; TNF-R1 — tumor necrosis factor-receptor 1; TNF-R2 — tumor necrosis factor-receptor 2.

## Data Availability

Requests to access datasets should be directed to the last author at helle.hvilsted.nielsen@rsyd.dk. The data are not publicly available because of ethical restrictions.

## Supplementary Materials

The following supporting information can be downloaded at https://www.mdpi.com/article/10.3390/ijms25031592/s1.
