# Supplementary material for: Performance Measures and Plasma Biomarker Levels in Patients with Multiple Sclerosis after 14 Days of Fampridine Treatment: An Explorative Study
Source: Int J Mol Sci. 2024 Jan 27;25(3):1592. doi: 10.3390/ijms25031592 (PMC10855557; doi:10.3390/ijms25031592)
Supplement: Supplementary file 1 [file ijms-25-01592-s001.zip › Supplementary Figure 1.pdf]

Supplementary Figure. *Inter-individual differences in cytokine levels between visit 1 and visit 2 in patients with multiple sclerosis, after 14 days of fampridine treatment.*

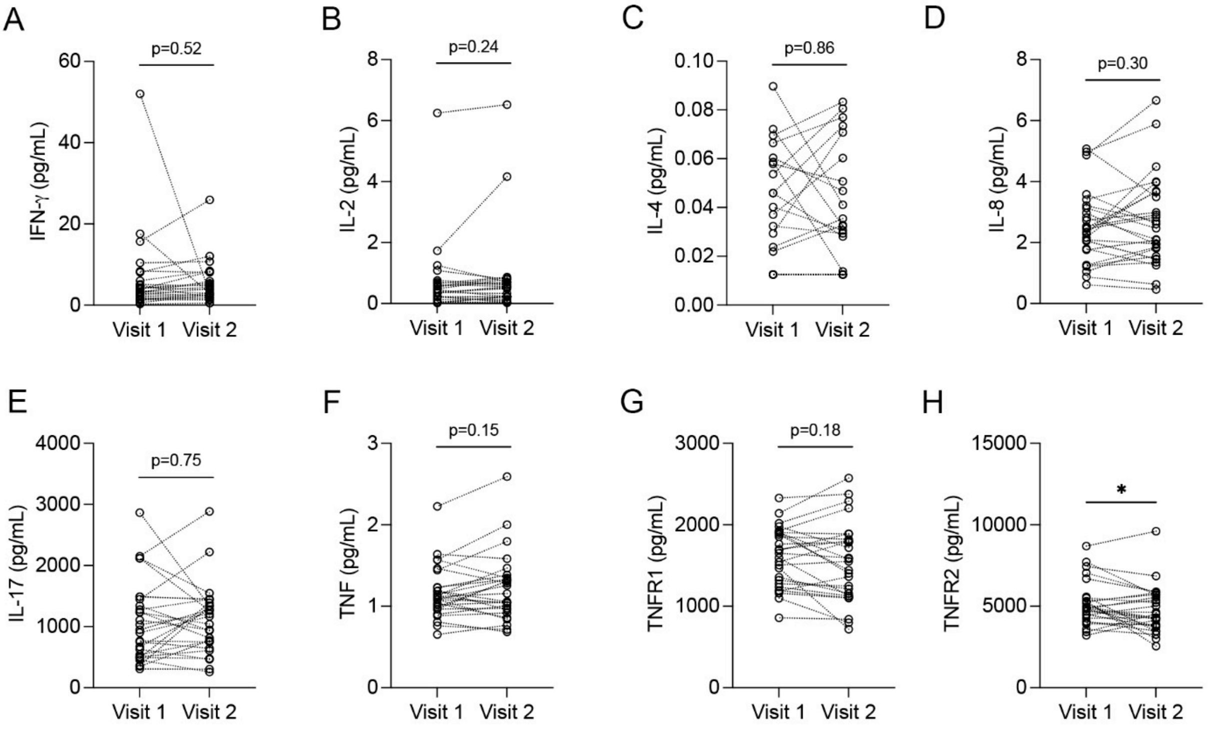

Supplementary Figure S1. Inter-individual differences in cytokine levels between visits. A: IFN-γ; B: IL-2; C:IL-4; D: IL-8; E: IL-17; F: TNF-α; G: TNFR1; H: TNFR2. Abbreviations: IFN-γ: Interferon gamma; IL: Interleukin; NFL: Neurofilament light; TNF: Tumor necrosis factor; R1: Receptor 1; R2: Receptor 2.
