# Supplementary material for: Performance Measures and Plasma Biomarker Levels in Patients with Multiple Sclerosis after 14 Days of Fampridine Treatment: An Explorative Study
Source: Int J Mol Sci. 2024 Jan 27;25(3):1592. doi: 10.3390/ijms25031592 (PMC10855557; doi:10.3390/ijms25031592)
Supplement: Supplementary file 1 [file ijms-25-01592-s001.zip › Supplementary tables_study 3.pdf]

Supplementary: *Tables of missing data at the two visits and of correlations between change in biomarkers in plasma and change in performance measures in patients with multiple sclerosis, after 14 days of fampridine treatment*

Table S1a. Overview of missing data at T_0 for the individual variables in patients with MS, n=27

Measure	Variable	Reason for missing	Missing data n (%)
**Biomarkers**			
	IL-2	Non-detectable	1 (3.7)
	IL-4	Non-detectable	1 (3.7)
	IL-8		0
	IL-17	Non-detectable	5 (18.5)
	IFN-γ		0
	TNF-α		0
	TNFR1		0
	TNFR2		0
	NFL		0
	GFAP		0
**Performance measures**			
	T25FW		0
	SSST		0
	2MWT		0
	9HPT		0
	MSWS-12	Technical problems	1 (3.7)

Table S1b. Overview of missing data at T_1 for the individual variables, n=27

Measure	Variable	Reason for missing	Missing data n (%)
**Biomarkers**			
	IL-2	Non-detectable	5 (18.5)
	IL-4	Non-detectable	1 (3.7)
	IL-8		0
	IL-17	Non-detectable	3 (11.1)
	IFN-γ		0
	TNF-α		0
	TNFR1	Non-detectable	1 (3.7)
	TNFR2		0
	NFL		0
	GFAP		0
**Performance measures**			
	T25FW		0
	SSST		0
	2MWT	Fatigue/tired	1 (3.7)
	9HPT		0
	MSWS-12	Technical problems	2 (7.4)

2MWT: Two-minute Walk Test; 9HPT: Nine Hole Peg Test; GFAP: glial fibrillary acidic protein; IFN: interferon gamma; IL: interleukin; IQR: interquartile range; MSWS-12: 12-Item Multiple Sclerosis Walking Scale; n: number; NFL: neurofilament light; R1: receptor 1; R: receptor 2; SD: standard deviation; SSST: Six Spot Step Test; T25FW: Timed 25-Foot Walk; TNF: tumor necrosis Factor.

Table S2. Correlations between change in biomarkers in plasma and change in performance measures in PwMS, after 14 days of fampridine treatment

**Correlation variables**	**Number (n)**	**Spearman's ρ (rho)**	**Correlation 95% Bca**	***p*-value**
**Correlations (change IFN-γ)**				
Change MSWS-12	25	-0.0543	[-0.4779 ; 0.3894]	0.7965
Change 9HPT	27	-0.1374	[-0.5441 ; 0.2953]	0.4944
Change SSST	27	-0.1789	[-0.5858 ; 0.2897]	0.3719
Change T25FW	27	-0.4559	[-0.7282 ; -0.0642]	**0.0168**
Change 2MWT	26	0.2205	[-0.1651 ; 0.6321]	0.2790
**Correlations (change IL-2)**				
Change MSWS-12	19	-0.0264	[-0.5205 ; 0.4785]	0.9145
Change 9HPT	21	-0.1364	[-0.6250 ; 0.4010]	0.5555
Change SSST	21	0.5179	[0.0387 ; 0.8166]	**0.0162**
Change T25FW	21	0.4646	[-0.0490 ; 0.8115]	**0.0339**
Change 2MWT	20	-0.1354	[-0.6268 ; 0.4400]	0.5693
**Correlations (change IL-4)**				
Change MSWS-12	23	0.0343	[-0.3891 ; 0.4843]	0.8764
Change 9HPT	25	0.0901	[-0.5056 ; 0.3719]	0.6685
Change SSST	25	-0.0740	[-0.4658 ; 0.3803]	0.7252
Change T25FW	25	-0.1705	[-0.5009 ; 0.2418]	0.4150
Change 2MWT	24	-0.1335	[-0.5066 ; 0.2535]	0.5340
**Correlations (change IL-8)**				
Change MSWS-12	25	-0.1988	[-0.5759 ; 0.2672]	0.3407
Change 9HPT	27	-0.1838	[-0.5398 ; 0.2239]	0.3588
Change SSST	27	-0.4057	[-0.6963 ; 0.0607]	**0.0357**
Change T25FW	27	-0.3979	[-0.6675 ; -0.0344]	**0.0398**
Change 2MWT	26	0.1234	[-0.3202 ; 0.4491]	0.5481
**Correlations (change IL-17)**				
Change MSWS-12	19	0.4605	[-0.7713 ; -0.0037]	**0.0473**
Change 9HPT	21	0.1429	[-0.3533 ; 0.6493]	0.5367
Change SSST	21	-0.5805	[-0.8018 ; -0.1264]	**0.0058**
Change T25FW	21	-0.3308	[-0.6960 ; 0.1848]	0.1429
Change 2MWT	20	0.1925	[-0.4217 ; 0.6950]	0.4162
**Correlations (change TNF-α)**				
Change MSWS-12	25	0.0975	[-0.2795 ; 0.4672]	0.6430
Change 9HPT	27	-0.2006	[-0.5633 ; 0.1649]	0.3158
Change SSST	27	-0.0171	[-0.4088 ; 0.4139]	0.9325
Change T25FW	27	-0.4159	[-0.7173 ; 0.1056]	**0.0309**
Change 2MWT	26	0.0195	[-0.3937 ; 0.4123]	0.9247
**Correlations (change TNFR1)**				
Change MSWS-12	24	-0.1224	[-0.5477 ; 0.4085]	0.5688
Change 9HPT	26	0.0865	[-0.3753 ; 0.5019]	0.6744
Change SSST	26	0.0947	[-0.3534 ; 0.5114]	0.6453
Change T25FW	26	-0.0759	[-0.4459 ; 0.3354]	0.7124
Change 2MWT	25	-0.1323	[-0.5160 ; 0.2549]	0.5284
**Correlations (change TNFR2)**				
Change MSWS-12	25	-0.0516	[-0.4760 ; 0.4194]	0.8064
Change 9HPT	27	0.2684	[-0.2145 ; 0.6447]	0.1759
Change SSST	27	0.0018	[-0.4420 ; 0.4480]	0.9928
Change T25FW	27	-0.1857	[-0.5624 ; 0.2622]	0.3538
Change 2MWT	26	0.1583	[-0.2853 ; 0.5429]	0.4399
**Correlations (change NFL)**				

Change MSWS-12	25	-0.3271	[-0.6865 ; 0.2016]	0.1105
Change 9HPT	27	0.2155	[-0.2330 ; 0.5988]	0.2803
Change SSST	27	-0.2421	[-0.6057 ; 0.1864]	0.2237
Change T25FW	27	-0.4086	[-0.7255 ; 0.0228]	**0.0343**
Change 2MWT	26	0.2581	[-0.1419 ; 0.5907]	0.2030
**Correlations (change GFAP)**				
Change MSWS-12	25	-0.3506	[-0.6465 ; 0.1452]	0.0857
Change 9HPT	27	0.1209	[-0.3482 ; 0.5051]	0.5480
Change SSST	27	-0.1526	[-0.5545 ; 0.3037]	0.4472
Change T25FW	27	-0.2098	[-0.5571 ; 0.2183]	0.2936
Change 2MWT	26	0.2602	[-0.1474 ; 0.5614]	0.1993

Correlations are expressed through Spearman's ρ (rho) and 95% BCa followed by a p-value. The 95% Bca written in red include 0. Abbreviations: 2MWT: Two-minute Walk Test; 9HPT: Nine Hole Peg Test; 95% BCa: bias-corrected, accelerated bootstrap confidence interval; GFAP: glial fibrillary acidic protein; IFN: interferon gamma; IL: interleukin; IQR: interquartile range; MSWS-12: 12-Item Multiple Sclerosis Walking Scale; n: number; NFL: neurofilament light; PwMS: patients with multiple sclerosis; R1: receptor 1; R2: receptor 2; SD: standard deviation; SSST: Six Spot Step Test; T25FW: Timed 25-Foot Walk; TNF: tumor necrosis Factor.
